# Physicochemical Properties of (La,Sr)CoO_3_ Thick Films on Fe-25Cr Steel under Exposure to SOFC Cathode Operating Conditions

**DOI:** 10.3390/ma17153791

**Published:** 2024-08-01

**Authors:** Janusz Prażuch, Michał Pyzalski, Daniel Fernández González, Tomasz Brylewski

**Affiliations:** 1 Faculty of Materials Science and Ceramics, AGH University of Krakow, al. Mickiewicza 30, 30-059 Krakow, Poland; prazuch@agh.edu.pl (J.P.); michal.pyzalski@agh.edu.pl (M.P.); 2 Centro de Investigación en Nanomateriales y Nanotecnología (CINN), Avda. de la Vega, 4-6, 33940 El Entrego, Spain; d.fernandez@cinn.es

**Keywords:** SOFC interconnects, La_0.6_Sr_0.4_CoO_3_ film, Fe-25Cr ferritic steel, screen printing, microstructure, electrical conductivity

## Abstract

La_0.6_Sr_0.4_CoO_3_ (LSC) coatings with a thickness of 50–100 µm were deposited on Fe-25Cr ferritic stainless steel (DIN 50049) via screen printing. The required suspension had been prepared using fine LSC powders synthesised using EDTA gel processes. In its bulk form, the LSC consisted entirely of the rhombohedral phase with space group *R-3c*, and it exhibited high electrical conductivity (~144 S·cm^−1^). LSC-coated steel was oxidised in air at 1073 K, i.e., under conditions corresponding to SOFC cathode operation, for times of up to 144 h. The in situ electrical resistance of the steel/La_0.6_Sr_0.4_CoO_3_ layered system during oxidation was measured. The products formed on the samples after the oxidation reaction resulting from exposure to the corrosive medium were investigated using XRD, SEM-EDS, and TEM-SAD. The microstructural, nanostructural, phase, and chemical analysis of films was performed with a focus on the film/substrate interface. It was determined that the LSC coating interacts with the oxidised steel in the applied conditions, and a multi-layer interfacial zone is formed. Detailed TEM-SAD observations indicated the formation of a main layer consisting of SrCrO_4_, which was the reaction product of (La,Sr)CoO_3_, and the Cr_2_O_3_ scale formed on the metal surface. The formation of the SrCrO_4_ phase resulted in improved electrical conductivity of the investigated metal/ceramics layered composite material, as demonstrated by the low area-specific resistance values of 5 mΩ·cm^2^, thus making it potentially useful as a SOFC interconnect material operating at the tested temperature. In addition, the evaporation rate of chromium measured for the uncoated steel and the steel/La_0.6_Sr_0.4_CoO_3_ layered system likewise indicates that the coating is capable of acting as an effective barrier against the formation of volatile compounds of chromium.

## 1. Introduction

Electrical energy production and its storage without any negative impact on the natural environment has become a serious challenge facing humanity nowadays when we can observe so many critical changes to the Earth’s systems caused by extreme weather events due to greenhouse gas emissions. One of the most challenging solutions in the production of clean energy is the use of SOFCs, which consume hydrogen or carbon monoxide [1,2,3,4,5]. Continued effort in research on the improvement of the usable properties of SOFCs is required in order to meet the anticipated technical and economic values. This especially regards metallic interconnects. Planar interconnects allow for the simple joining of individual SOFCs into a stack, whose working voltage and power are proportional to the number of cells [5].

An interconnector with planar geometry is in the form of a plate, several mm thick, with channels on both planar sides that are used to supply gas reagents to both electrodes, anode and cathode. Interconnects are typically made of metallic steel [5,6,7,8,9,10,11,12,13]. Such steels should fulfil the following requirements: a coefficient of thermal expansion similar to that of other SOFC components; structural stability; stable and appropriate mechanical properties at high temperatures; low value and low growth with time area specific resistance (ASR); and chemical inertness towards other SOFC components [5,6,7,8,9,10,11,12,13]. High-chromium ferritic steel is currently the only commercially viable alloy metal for SOFC interconnects, despite the fact that it does not fully satisfy the required conditions. High-chromium steels oxidise to a layered oxide scale built mainly of Cr_2_O_3_ in all oxidising environments [6,7,8,10,11,12]. The thickness of the oxide layer, exhibiting semiconducting properties, continuously increases with time in the SOFC’s operating conditions, resulting in an increase in the electrical resistance of the whole steel/scale system [6,7,8]. Additionally, chromium evaporating from the steel substrate reacts with the cathode material, leading to deterioration of the electrochemical efficiency of the fuel cell [14,15,16,17]. This effect would be disadvantageous to the operation efficiency of a cell with a Fe-Cr steel interconnect [5]. An inexpensive and simple method is proposed to stop this harmful effect and preserve the desired useful properties of the metallic interconnect by coating it with a ceramic protective and conductive layer of lanthanum strontium cobaltite (La,Sr)CoO_3_ (LSC) using the screen printing method. Screen printing is an easy and well-established printing and coating method, and in this work, it is proposed to modify the Fe-25Cr steel surface. The (La,Sr)CoO_3_ coating is of particular interest and can be deposited by various methods [18,19,20,21,22,23]. However, the mechanism of this improvement has not been fully explained yet due to the lack of detailed microstructure observations.

The La_1-x_Sr_x_CoO_3-δ_ compound is a mixed ion-electron conductor with predominant electronic transport [24,25,26,27,28,29]. The crystal structure and electrical properties of this compound are complex and depend both on the Sr amount (x) and temperature [26]. At low temperatures and with x up to 0.2, it has a distorted rhomboederal crystal structure, which becomes ordered with the temperature increase. Due to the hopping of small polarons between Co^3+^ and Co^4+^ ions in the cobalt sublattice of the La_1-x_Sr_x_CoO_3-δ_ compound, it exhibits semiconducting properties [25]. For higher amounts of Sr, x ≥ 0.3, this compound crystallises in the cubic structure, and its conductivity, decreasing with temperature starting from room temperature, exhibits metallic character practically at all temperatures [25]. The maximum value of conductivity occurs at x = 0.4, and it can reach 740 S·cm^−1^ at 1073 K in air [24]. Strontium dopant in the lanthanum cobaltite oxide leads to the formation of electronic defects in the form of Co^4+^, which implies the following chemical formula of the oxide—La^3+^_1-x_Sr^2+^_x_Co^3+^_1-x_Co^4+^_x_O_3-δ_ [24,25]. For x ≥ 0.4, especially at high temperatures, the predominant point defects in the LSC are the positively charged oxygen vacancies [25]. Their presence gives the LSC high ionic conductivity, e.g., 5.6 × 10^−3^ S·cm^−1^ to 1.2 × 10^−2^ S·cm^−1^ at 700 °C, suitable for SOFC cathode material [30,31]. The LSC oxide can be used for the construction of intermediate-temperature solid oxide fuel cells (IT-SOFCs) operating in the range of 500–700 °C, which demonstrate that excellent ionic and electronic conductivities extend the triple-phase boundary (TPB) region from the cathode–electrolyte interface to the bulk of the cathode [32].

It is noteworthy that studies on the application of the LSC perovskite as an efficient oxygen electrode material in the form of partially amorphous La_0.6_Sr_0.4_CoO_3-δ_ for low-temperature solid oxide fuel cells (LT-SOFCs) operating at 400 °C are carried out [33]. Chemical and phase similarity between the LSC electrode and the LSC coating on the ferritic steel interconnect could allow for long-term, stable integration of both SOFC components.

In the first experimental stage, La_0.6_Sr_0.4_CoO_3_ powder was synthesised by EDTA gel processes using ethylene-diamine-tetra-acetic acid (EDTA) as a chelating agent and metal precursors. The obtained LSC material was characterised both in powder form and as sintered ceramics. In the next stage, investigations were performed on the Fe-25Cr ferritic steel covered with the La_0.6_Sr_0.4_CoO_3_ film by the screen printing method. The coated and uncoated steels were oxidised in air at 1073 K. The in situ electrical resistance of the steel/La_0.6_Sr_0.4_CoO_3_ layered system during oxidation was measured. The post-oxidation investigation of the phase and chemical composition, morphology, and nanostructure of the coating, with particular attention to the metal substrate/coating interface, was performed and discussed in terms of applicability in SOFCs.

**Figure 1:**
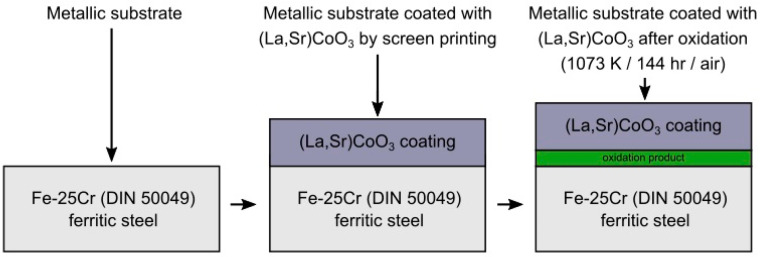
Graphical summary of the research concept related to the development of a layered system consisting of ferritic steel Fe-25Cr and an (La,Sr)CoO_3_ coating.

Figure 1 shows a graphical summary of the research concept involving the surface modification of Fe-25Cr ferritic steel using a (La,Sr)CoO_3_ protective–conductive coating for application in SOFC interconnects. After high-temperature oxidation in air, an intermediate layer was formed at the steel/coating interface, labelled “oxidation product”, which was composed both of thermally grown oxide (TGO) on the metal surface and a product of reaction between the TGO layer, the steel substrate, and the coating due to inward and outward diffusion of ions.

## 2. Materials and Methods

### 2.1. Preparation of (La,Sr)CoO_3_ Powders by EDTA Gel Processes

The synthesis of La_0.6_Sr_0.4_CoO_3_ powder was performed by EDTA gel processes using EDTA as a complexing agent of metal cations in a water solution. The versenate precursors were prepared from the Reagent (AR) grade reagents obtained from Sigma Aldrich (Saint Louis, MO, USA): La(NO_3_)_3_·6H_2_O (99.99%), Sr(NO_3_)_2_ (99.9%), Co(NO_3_)_3_·6H_2_O, and EDTA—ethylene-diamine-tetra-acetic acid as a complexing agent of metal cations in a water solution of La, Sr, and Co nitrate. A total of 0.5 M solutions were prepared and sequentially mixed in adequate proportions of La:Sr:Co = 0.6:0.4:1.0. The resultant solution was mixed with 0.1 M EDTA in a ratio of 1 mole EDTA to 1 mole metal cation. The pH of 8 was maintained by the dropwise addition of ammonia.

During liquid precursor synthesis, stable complex ions are formed according to the simplified chemical reactions:

(1) $$H_{4}ETA+4NH_{3}\to EDTA^{-4}+4NH_{4}^{+}$$

(2) $$Co{\left(NO_{3}\right)}_{2}+6NH_{3}\to {\left[Co{\left(NH_{3}\right)}_{6}\right]}^{2+}+2NO_{3}^{-}$$

(3) $$EDTA^{4-}+M{\left(NO_{3}\right)}_{3}\to {\left[M\left(EDTA\right)\right]}^{-}+3NO_{3}^{-}$$

where $M={La}^{3+}$

(4) $$EDTA^{4-}+A{\left(NO_{3}\right)}_{2}\to {\left[A\left(EDTA\right)\right]}^{2-}+2NO_{3}^{-}$$

where $A={Co}^{2+},Sr^{2+}$

As reaction (1) shows, ammonium in solution shifts the position of equilibrium of EDTA dissociation towards complex EDTA^4-^ ions, which subsequently bind to La^3+^, Co^2+^, and Sr^2+^ ions present in the solution to form appropriate, soluble in water, stable complex ions of these metals (reactions 3 and 4). At the same time, in the solution of EDTA with ammonium and cobalt(II) nitrate(V), ammonium is bound to Co^2+^ ions to form complex hexaamminecobalt(II) ions (reaction (2)). This complex formation reaction prevents Co(OH)_2_ precipitation.

The resulting precursor solution was heated in vacuum at temperatures between 333 and 383 K, transforming from sol to gel, which was subsequently dried at 393 K for 10 h and then subjected to pyrolysis in air at 773 K for 1 h. Thermal decomposition of versenate yielded fine black-coloured pyrolyzate, which was finally calcined in air at 1273 K for 25 h. This material was further ground by ball milling in a liquid medium and finally ultrasonically fragmented, thus obtaining the LSC powder.

### 2.2. Sintering of (La,Sr)CoO_3_ Bulk Sample

The LSC powder was uniaxially pressed at a pressure of 50 MPa to produce 10 mm-diameter and 2–4 mm-thick green compacts, which were sintered without load in air at 1473 K for 12 h on the YSZ substrates. The sintering stage was followed by cooling at a rate of 2.5 K·min^−1^ down to room temperature.

### 2.3. Composition of (La,Sr)CoO_3_ Paste and Its Deposition on Steel Substrate by Screen Printing Method and Oxidation Process Procedure

Fe-25Cr high chromium ferritic steel (17153.2 CSN EN 10204 3.1 B (DIN 50049) Valcovny Plechu a.s. Frydek-Mistek, Czech Republic), performing the function of the SOFC metallic interconnect, was used in this study. The chemical nominal composition of this steel is shown in Table 1. The Fe-25Cr steel specimens were in the form of 20 mm × 10 mm × 1 mm coupons. No additional heat treatment was performed, and the steel was used as received.

Screen printing was used to deposit the LSC thick layer onto the steel substrate. La_0.6_Sr_0.4_CoO_3_ paste for screen printing was prepared from a 5:1 mass ratio mixture of the LSC powder and organic binder, which was a 5 wt.% solution of ethylocellulose (Fluka-Bio Chemika) in terpineol (Terpineol anhydrous—Fluka Chemika). The paste components were mixed and homogenised in a closed system for 10 h. The required dynamic viscosity of 45–50 P, suitable for screen printing, was adjusted by adding terpineol. Thick films were deposited on steel substrates on the abraded surfaces by screen printing using a 100 mesh screen. The films were about 50–100 μm in thickness. To obtain coatings of different thicknesses, printing was repeated two to three times.

The organic components were removed entirely from the paste during the heating of the coated specimens in air up to 623 K at a1 K·min^−1^ heating rate and maintaining that temperature for 1 h during the initial step of the oxidation process. Oxidation of Fe-25Cr steel covered with (La,Sr)CoO_3_ coating was performed in air (pO_2_ = 0.21 atm) at 1073 K for 144 h. Cooling was performed at a rate of 2 K·min^−1^. A non-coated steel coupon oxidised and cooled in the same above-mentioned conditions played the role of a reference sample.

### 2.4. Analysis Method

The chemical composition of the obtained powder was analysed by flame atomic absorption spectrometry (AAS) using a Pye Unicum SP90B spectrometer (Pye Ltd., Cambridge, UK) and an air-acetylene flame. The granulometric distribution of the obtained powder was measured by the laser diffraction method (LD) using the Mastersizer 2000 apparatus of Malvern Instruments (Malvern Panalytical, Malvern, UK). Malvern Application v. 5.60 software was used for data analysis (Malvern Panalytical, Malvern, UK). The specific surface area of the powder was determined using a Micromeritics ASAP 2010 BET Surface Area Analyzer (Micromeritics GmbH, Unterschleißheim, Germany) through nitrogen gas adsorption under liquid nitrogen conditions (i.e., 77 K). The average particle diameter of the powder was evaluated from the measured BET surface area and knowledge of the material density (on the premise that particles are spherical and non-porous) using the relation [34]:

(5) $$d_{BET}=\frac{6}{\rho_{XRD}\cdot S}$$

where S is the specific surface area of the sample [m^2^·g^−1^], ρ_XRD_ is its X-ray density [g·m^−3^], and d_BET_ is the BET particle diameter [m].

Scanning electron microscopy (SEM) using FEI Nova 200 nanoSEM (FEI Europe Company, Eindhoven, The Netherlands) coupled with an EDAX Genesis XM X-ray microanalysis system based on the EDAX Saphire Si(Li) EDS (EDAX, Tilburg, The Netherlands) detector was used to provide information both on the morphology and chemical composition of sintered powders, surface and cross-section morphology and microstructure, as well as the chemical composition of steel/coating and steel/oxide scale layered samples. The powder morphology and chemical composition were analysed using an Apreo 2S high-resolution scanning electron microscope (Thermo Fisher Scientific, Waltham, MA, USA) and the software Phenom Prosuite (Thermo Fisher Scientific, Waltham, MA, USA, https://www.thermofisher.com/us/en/home/electron-microscopy/products/software-em-3d-vis/prosuite-software.html, accessed on 26 June 2024) for collecting the EDS spectra.X-ray diffraction (XRD) was used to identify crystalline phases present in the compact and non-compact powder, as well as steel/coating and steel/oxide scale samples. For this purpose, the Panalytical X′ Pert Pro PW 3710 diffractometer (Panalytical, Almelo, The Netherlands) was used with CuKα monochromatic radiation. The HighScore Plus computer software v.5.1 and the standard PCPDFWIN v.2.3 data set were used to identify the phase composition of the tested materials (Panalytical, Almelo, The Netherlands). The mass fractions of individual phases, as well as their lattice parameters, were determined via Rietveld refinement. X-ray measurements allowed us to evaluate the size of the grains in the powder using the Scherrer equation [35].

The bulk density of sintered powders, and thus the degree of sintering, was determined by hydrostatic weighing. The total porosity (P_c_) of the sintered compact was computed using Equation (6) [36]:

(6) $$P_{c}=\left(1-\frac{d_{a}}{d_{XRD}}\right)\cdot 100\%$$

where d_a_ is the apparent density [g·cm^−3^] and d_XRD_ is the theoretical density [g·cm^−3^]. The theoretical density of the La_0.6_Sr_0.4_CoO_3_ sinter, calculated based on crystallographic data, was 6.5852 g·cm^−3^.

The steel/coating interface was analysed in detail at the nanoscale by conventional transmission electron microscopy (TEM), performing structural determination by means of the selected area electron diffraction (SAED) technique. A Philips CM20 transmission electron microscope operating at 200 kV was applied in this TEM study (Philips, Eindhoven, The Netherlands).

The rate at which chromium evaporates from the studied samples was measured using an apparatus with a design proposed by Kurokawa et al. [37]. This experiment was conducted at 1073 K for 72 h in air–water vapour gas mixture flow with a rate of 120 mL·min^−1^ and water vapour partial pressure p(H_2_O) = 9849 Pa. A detailed description of the experimental setup used for the measurement of the Cr evaporation rate and the initial test aimed at establishing the dependence between the mass of chromium evolved during the evaporation of pure chromia and the flow rate of carrier gas is given in Refs. [38,39]. The measurements of the evaporation rate of Cr were performed for unmodified Fe-25Cr steel as well as the Fe-25Cr/(La,Sr)CoO_3_ layered systems; each of these materials underwent 144 h of oxidation in air at 1073 K. After each experiment, the glass parts of the apparatus were thoroughly cleansed with destilled water. Nitric acid was added to the water solution containing chromium ions, and the obtained acidic solution was concentrated to a given volume (20 cm^3^) by solvent evaporation. The Cr content was determined by means of the ICP-OES OPTIMA 7300 DV (Perkin Elmer, Waltham, MA, USA). The accuracy of this determination was 2%.

A four-point probe was used to measure the resistivity of sintered compacts and steel/coating samples using Pt electrodes. The electrical measurements were conducted in laboratory air. Sintered bodies were tested with a 10 mA current and a temperature range of 673 K to 1098 K, whereas the steel/coating was tested at 1073 K using a current of 300 mA. The resistivity test stand used in this study is described elsewhere [40].

Knowledge of the measured resistivity and geometrical dimensions of the sintered compacts allowed us to calculate their electrical conductivity using the equation [41]:

(7) $$\sigma_{m}=\frac{L}{A\cdot R}$$

where R is the sample resistivity [Ω], L is the sample thickness [cm], and A is the area of the Pt electrodes [cm^2^]. The obtained electrical conductivity data were adjusted for porosity. For this purpose, the Bruggeman model was applied according to the following formula [42]:

(8) $$\sigma =\sigma_{m}\cdot \frac{1}{{\left(1-p\right)}^{3/2}}$$

where σ is the conductivity after adjusting for porosity [S·cm^−1^], σ_m_ is the measured conductivity [S·cm^−1^], and p is the sinter porosity [-].

Area-specific resistance (ASR) was calculated for the steel/coating sample from the following equation [6,12]:

(9) $$ASR=\frac{R\cdot B}{2}$$

where R is the electrical resistance [Ω] and B is the surface area of the Pt layer [cm^2^].

## 3. Results

### 3.1. Physicochemical Properties of (La,Sr)CoO_3_ Powder

Based on the results of complex thermal analysis and analysis of the phase composition of the gel precursor at different stages of its physico-chemical changes during heating, the following calcination conditions leading to the powder of the established (La,Sr)CoO_3_ composition and fine crystalline structure were suggested: temperature of 1273 K, time of 25 h, and air as a gas atmosphere.

X-ray diffraction of the obtained LSC powder allowed for the identification of only one rhombohedral LSC phase and thus confirmed its single-phase composition and the absence of unreacted substrates. The mean size of crystallites was determined to be as small as 31 nm using the Scherrer equation. This result, together with the result of granulometric analysis, leads to the conclusion that the proposed EDTA gel processes enable the manufacturing of fine-grained powder with possibly high reactivity, which could favour the formation of a dense and well-sintered LSC coating on the metallic substrate.

To determine deviation from stoichiometry of the LSC composition after calcination, its chemical composition was analysed by atomic absorption spectrometry (AAS), and the following mole percentages for La, Sr, and Co were obtained: 0.60, 0.43, and 1.07%, respectively, which is close to the established stoichiometry: La:Sr:Co = 0.6:0.4:1.

**Figure 2:**
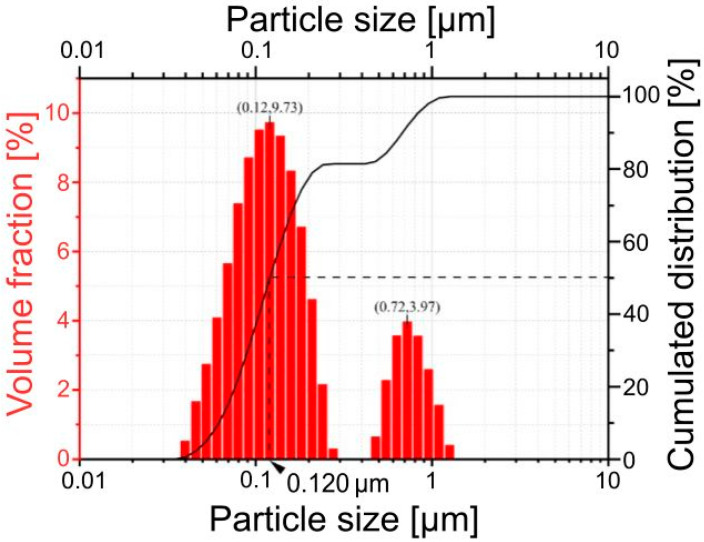
Particle size distribution and cumulated distribution of the (La,Sr)CoO_3_ powder after calcination at 1273 K for 25 h in air.

Figure 2 presents particle size distribution results—the histogram and the cumulative distribution curve—while the detailed statistical information derived from the particle size distribution and the results of BET analysis for the (La,Sr)CoO_3_ powder after calcination at 1273 K for 25 h in air are presented in Table 2. From these results, it can be concluded that the prepared powder was characterised by a bimodal particle size distribution. The powder is composed of two grain populations with distinct sizes—the first peak of the bimodal PSD represents very fine grains, 0.03–0.3 μm, which are in the majority in the whole population; the second peak represents the grain agglomerates ranging in size from 0.4 to 1.5 μm.

**Figure 3:**
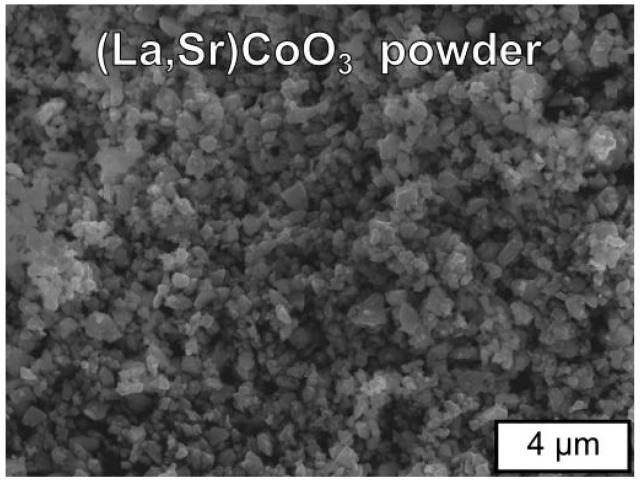
SEM micrograph of (La,Sr)CoO_3_ powder after calcination at 1273 K for 25 h in air.

Scanning electron microscopic (SEM) observations yielded information about the characteristics of the grains of the studied LSC powder. These grains had an irregular shape and a size of 0.02 to 0.2 μm and also formed agglomerates with a diameter of 0.5–1.3 μm (Figure 3).

Summarising the results of the grain size determination of the obtained calcined powder, one can conclude that both the analysis performed using SEM images (Figure 3) and the laser diffraction method (LD) (Table 2) provided very similar, comparable data.

### 3.2. Physicochemical Properties of (La,Sr)CoO_3_ Sinter

The degree of densification for the sintered La_0.6_Sr_0.4_CoO_3_ compact, being the measure of powder sinterability, was determined from the relative density and the total porosity. The relative density of a sample was calculated by dividing its apparent density (ρ) obtained from hydrostatic weighing in distilled water by the theoretical density (ρ_XRD_) calculated from the crystallographic data. The obtained values of apparent density, relative density, and total porosity for the La_0.6_Sr_0.4_CoO_3_ compact sintered at 1473 K in air for 12 h are collected in Table 3.

A high relative density of about 97% of the theoretical density of the sintered compact seems to imply a possibly high density of the sintered LSC coating on the metallic substrate.

Similarly to the powder, X-ray phase analysis of the La_0.6_Sr_0.4_CoO_3_ compact sintered at 1473 K in air for 12 h confirmed its single-phase composition. Rietveld numerical analysis indicated the presence of a rhombohedral phase with *R-3c* space group and the following lattice constants: a = b = 0.54359(12) nm and c = 1.32158(54) nm, comparable with the table data [24,25,26,27,28,29,30,31,32,33,34,35,36,37,38,39].

**Figure 4:**
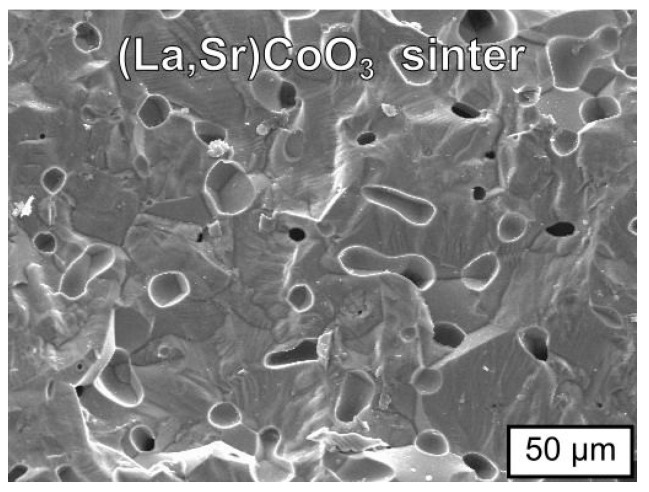
SEM micrograph of the fractured cross-section of the La_0.6_Sr_0.4_CoO_3_ compact sintered at 1473 K in air for 12 h.

Figure 4 shows the SEM morphology of the fractured cross-section of the La_0.6_Sr_0.4_CoO_3_ compact sintered at 1473 K in air for 12 h.

SEM morphology revealed a dense and coarse grain structure, with grain sizes between 15 and 50 µm and isolated pores. Such a result is in accordance with the determined degree of densification for the sintered compact (Table 3).

Electrical conductivity of the La_0.6_Sr_0.4_CoO_3_ compact sintered at 1473 K in air for 12 h was measured in the temperature range from 673 to 1098 K in air in the compound thermodynamic stability conditions, which was indicated by ex-situ analysis of the structure and chemical composition of the sample after electrical measurement, exhibiting preservation of both parameters, and additionally supported by the literature data [43].

**Figure 5:**
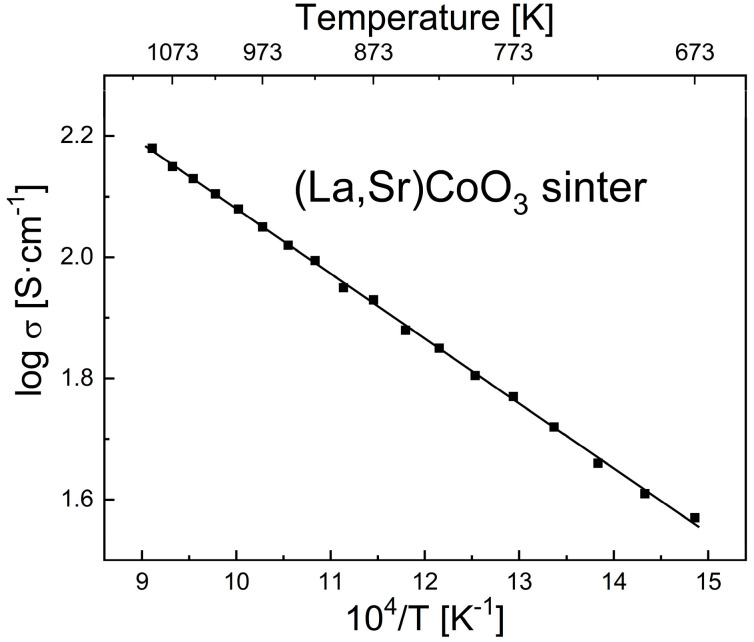
Electrical conductivity vs. temperature in the Arrhenius layout for the La_0.6_Sr_0.4_CoO_3_ compact sintered at 1473 K in air for 12 h.

Figure 5 shows electrical conductivity vs. temperature in the Arrhenius layout for the La_0.6_Sr_0.4_CoO_3_ compact sintered at 1473 K in air for 12 h. Calculations of the electrical conductivity of the tested sample include the porosity factor (Table 3) using Equation (8).

The value of the conductivity indicated a weak semiconducting property of the LSC sample, allowing for the conclusion that electrical conductance in this material is determined by the mechanism of small polarons, according to the equation [44,45]:

(10) $$\sigma =\frac{\sigma_{o}}{T}\cdot exp\frac{-E_{c}}{k\cdot T}$$

where σ_o_ is the pre-exponential factor, dependent on higher and lower valence ion concentrations and some other material parameters [Ω^−1^cm^−1^K^−1^], and E_c_ is the activation energy of small polaron movement (polaron migration enthalpy) [eV].

The measured electrical conductivity (σ) of 144.17 Ω^−1^·cm^−1^ at 1073 K was significantly lower than the literature value mentioned in the Introduction section [24].

Taking into account a linear relationship of the equation ln(σ·T) = f(1/T) [44,45], the activation energy of conductivity of 0.28 eV for the temperature range of 673–1094 K for the tested material was derived by the least-squares method. The error in the activation energy value did not exceed 0.01 eV.

### 3.3. Physicochemical Properties of the Coating (La,Sr)CoO_3_/Fe-25Cr Steel

In SOFC operating conditions at high temperatures at the steel/coating interface, physicochemical interaction between both materials may occur, e.g., interdiffusion and chemical reactions, leading to changes in chemical or/and phase compositions at the interface depending on the nature of the interaction. Due to chemical changes, new phases may be formed, typically in the form of an intermediate layer between the steel surface and the coat. Such a layer influences the adhesion of the coat to the metallic core and the operating parameters, e.g., electrical and mechanical, of the whole steel/coating system. Because of this, it is necessary to take a closer look at the interface of the tested Fe-25Cr steel/LSC coating systems after exposure to the simulated SOFC’s operating conditions in this work, i.e., at 1073 K in air for 144 h. The interfacial zone also plays an important role at the steel/oxide scale interface, where some intermediate phases may also be formed, typically in the layered structure, due to changes in activity of the oxidant and metallic substrate’s elements.

**Figure 6:**
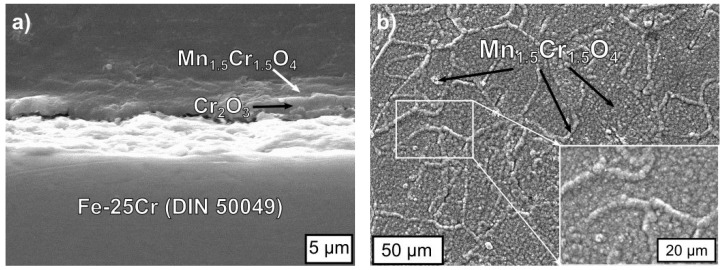
SEM images of the oxide scale formed on Fe-25Cr steel after oxidation for 144 h in air at 1073 K: (**a**) image from the polished taper cross-section and (**b**) image of the scale surface with higher magnification on the insert.

Figure 6 shows a SEM micrograph of the oxide morphology formed on the Fe-25Cr steel at 1073 K for 144 h in air. The oxide scales were duplex and composed of a Mn_1.5_Cr_1.5_O_4_ spinel in the outer part and of Cr_2_O_3_ in the inner region, the main oxidation product being chromia, which is seen in the cross-sections (Figure 6a) and confirmed by XRD analysis. The oxide scale was compact and fairly adherent to the metallic core. The chromia layer was built of columnar grains and was 2 μm thick, whereas the spinel layer was approximately 0.5 μm thick.

The surface of the scale, as presented in Figure 6b, was flat but with some thick nodular growths and string-like precipitations. The strings were about 4 μm wide, and the nodules were about 5–10 μm in diameter. These linear precipitations and the nodules were composed of Mn_1.5_Cr_1.5_O_4_, which is confirmed by EDS and XRD analyses and on the basis of the literature [46,47].

**Figure 7:**
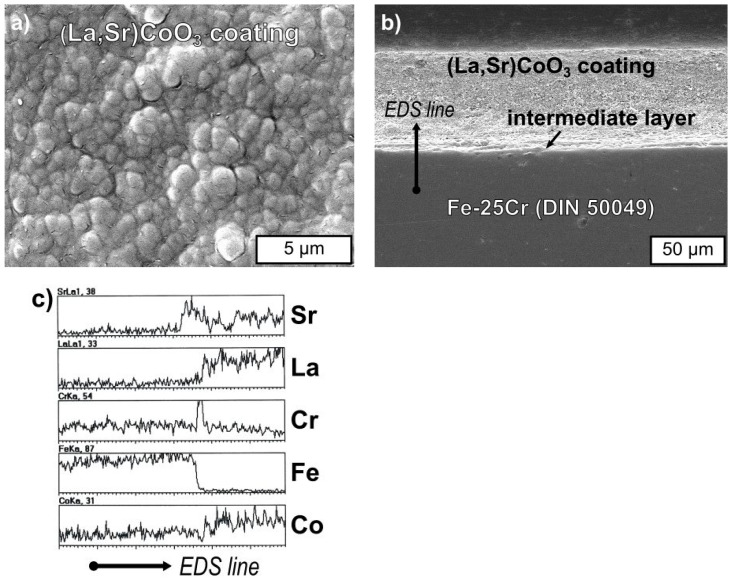
(**a**) SEM images of Fe-25Cr steel covered with La_0.6_Sr_0.4_CoO_3_ oxidised for 144 h in air at 1073 K; (**b**) images from the polished taper cross-section; (**c**) the EDS line scan runs along the black line in (**b**) across the metal/oxide interphase.

Figure 7a,b shows SEM micrographs of the oxide morphology formed on the Fe-25Cr steel coated with the (La,Sr)CoO_3_ layer at 1073 K for 144 h in air, showing the surface features (Figure 7a) and cross-section (Figure 7b). Figure 7c shows EDS linescan analysis for Sr, La, Cr, Fe, and Co along the line in Figure 7b across the steel/coating border.

SEM-EDS analysis revealed that the 100 μm-thick (La,Sr)CoO_3_ layer is compact and well adherent to the metallic substrate. It is composed of regular grains 0.5 μm–1.5 μm in size. Under the coating, a compact Cr_2_O_3_ layer was formed. EDS linescan analysis confirmed significant amounts of Cr and Sr in the bottom part of the oxide scales, close to the metal core/scales border. The concentration of La and Sr in the outer part of the coating is consistent with the La_0.6_Sr_0.4_CoO_3_ compound stoichiometry.

**Figure 8:**
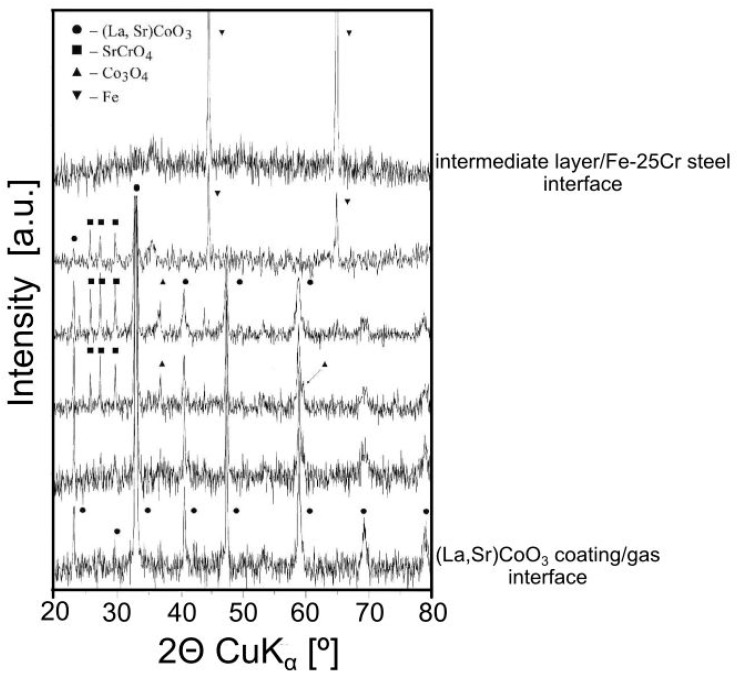
XRD patterns of (La,Sr)CoO_3_ film on Fe-25Cr steel after oxidation in air at 1073 K for 144 h for different depths from the gas/film to the film/metal interface.

A series of XRD patterns of the Fe-25Cr steel coated with the (La,Sr)CoO_3_ layer oxidised at 1073 K for 144 h in air were obtained after sequential thinning of the oxide layer on the steel substrate down to the metal/oxide interface (Figure 8).

The pattern from the outer surface of the coating confirmed that (La,Sr)CoO_3_ occurs as the main phase in the outer portion of the oxide layer. The patterns from the portion close to the metallic core revealed the presence of SrCrO_4_ and Co_3_O_4_. La_2_O_3_ and Co_3_O_4_ in the bottom part of the coating were also confirmed.

**Figure 9:**
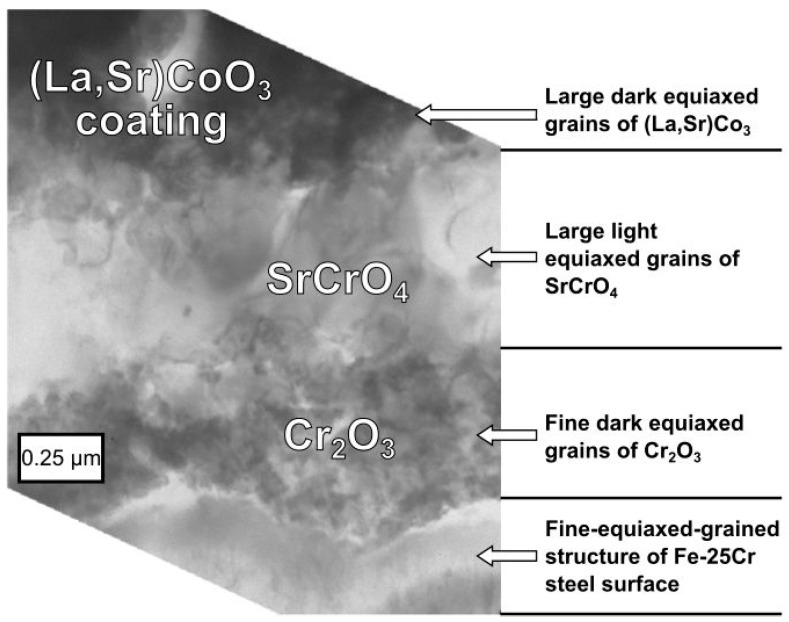
TEM cross-section micrograph of the multilayer metal/oxide interface between the (La,Sr)CoO_3_ coating and the Fe-25Cr substrate.

A detailed study of the metal substrate/oxide layer interface zone of the Fe-25Cr steel coated with the (La,Sr)CoO_3_ layer sample oxidised at 1073 K for 144 h in air was conducted by conventional transmission electron microscopy (TEM) both in the mode of imaging (bright fields and dark fields—BFs and DFs, respectively) and electron diffraction (selected area diffraction—SAD). Figure 9 shows a TEM image revealing the layered structure of the interface between the (La,Sr)CoO_3_ coating and the Fe-25Cr substrate. The deepest part of the analysed oxide area, in contact with the metallic core, consisted of Cr_2_O_3_ crystals.

**Figure 10:**
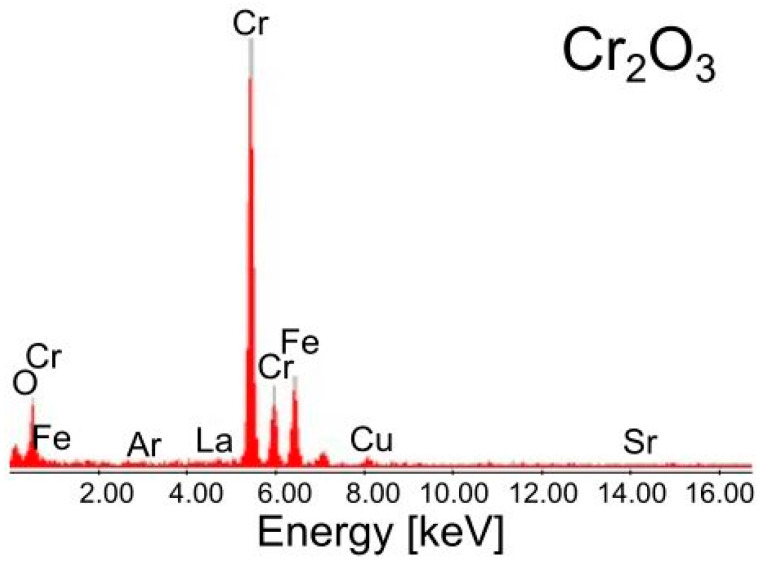
EDS spectrum of the chromia layer formed at the Fe-25Cr/(La,Sr)CoO_3_ interface.

An EDS spectrum of these grains (Figure 10) showed a weak oxygen peak due to the large thickness of the TEM foil at the point of analysis.

It may also be responsible for the presence of the Fe peak in the EDS spectrum of this inner chromia layer because of a larger analysis volume, possibly partially covering the steel substrate. Another possible explanation of this fact is the doping of Cr_2_O_3_ with Fe originating from the underlaying Fe-25Cr steel. Cr_2_O_3_ and Fe_2_O_3_ are isostructural oxides with similar radii of the cations (Cr^3+^ 75.5 pm (LK = 6); Fe^3+^ 69.0 pm (LK = 6 LS-low spin) [48]), and the oxides can form a continuous solid solution in the entire concentration range. Fe_2_O_3_ (4 mol%) doped Cr_2_O_3_ results in an increase in the chromia conductivity at 1073 K in air [49,50], which should favourably affect the electrical properties of the LSC-coated Fe-25Cr steel under SOFC operating conditions.

**Figure 11:**
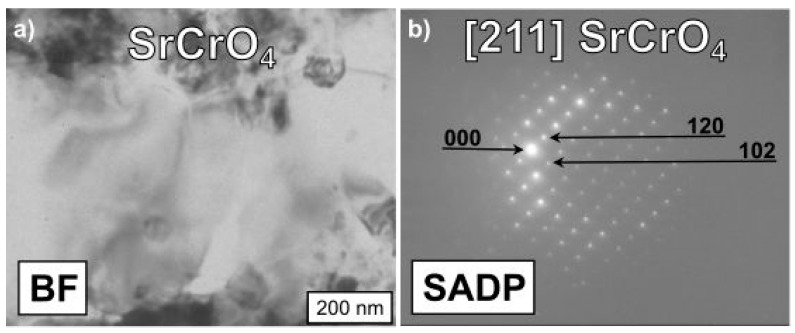
(**a**) TEM cross-section micrograph, and (**b**) SAD pattern with the [211] zone axis of the SrCrO4 layer.

The intermediate layer of approximately the same thickness as the underlying Cr_2_O_3_ layer, as identified by TEM-SAD, was built of large uniaxial grains (Figure 9) and identified by SAD as a crystalline SrCrO_4_ phase (Figure 11). Elemental EDS analysis of these grains exhibited mainly the presence of Sr and Cr. The outermost layer is composed of very large crystallites with an average size of about 0.5 μm in the La_0.6_Sr_0.4_CoO_3_ phase. Additionally, small amounts of the La_2_O_3_ phase were confirmed.

**Figure 12:**
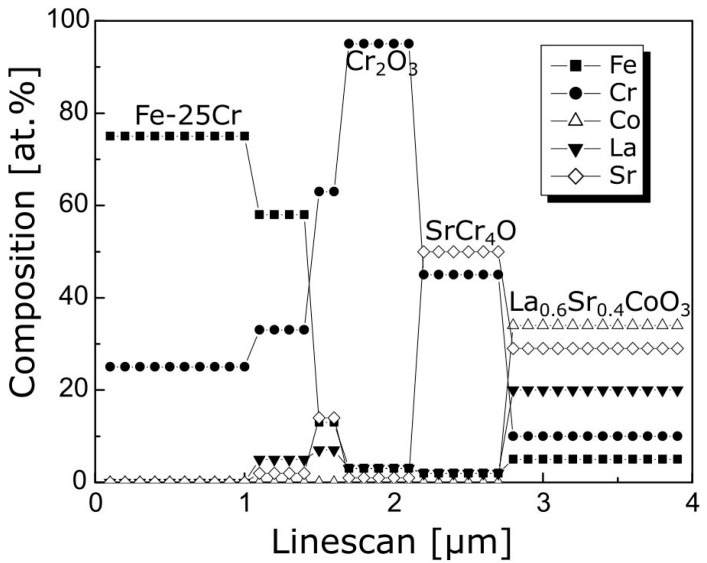
Quantitative EDS line scan analysis across the Fe-25Cr substrate/(La,Sr)CoO_3_ film interface shown in Figure 9.

Figure 12 illustrates chemical composition changes in the cross-section of the oxide layer formed on the Fe-25Cr steel coated with (La,Sr)CoO_3_ after oxidation at 1073 K for 144 h in air.

To sum up the TEM-SAD observations, one could conclude that during high-temperature oxidation of the Fe-25Cr/(La,Sr)CoO_3_ system, strontium diffuses to the grain boundaries between chromia grains grown at the initial stages of oxidation, thus forming the SrCrO_4_ phase, according to the following reaction (11):

(11) $$3{La}_{0.6}{Sr}_{0.4}CoO_{3}+0.6{Cr}_{2}O_{3}+0.3O_{2}↔1.2SrCrO_{4}+0.9{La}_{2}O_{3}+{Co}_{3}O_{4}$$

The driving force of this physicochemical process results from the high thermodynamic stability of the SrCrO_4_ oxide [51,52]. Reaction (11) is fully consistent with the Cr_2_O_3_−SrO phase diagram investigated by T. Negas and R. Roth [53].

It is expected that the formed SrCrO_4_ layer may improve the electrical properties of the Fe-25Cr/(La,Sr)CoO_3_ system, but from the other side, it may worsen resistance to high-temperature gas corrosion.

To evaluate the usefulness of the (La,Sr)CoO_3_ coating on Fe-25Cr steel in the construction of a SOFC interconnector, the area-specific resistance (ASR) was measured for the coated steel during oxidation at 1073 K in air.

**Figure 13:**
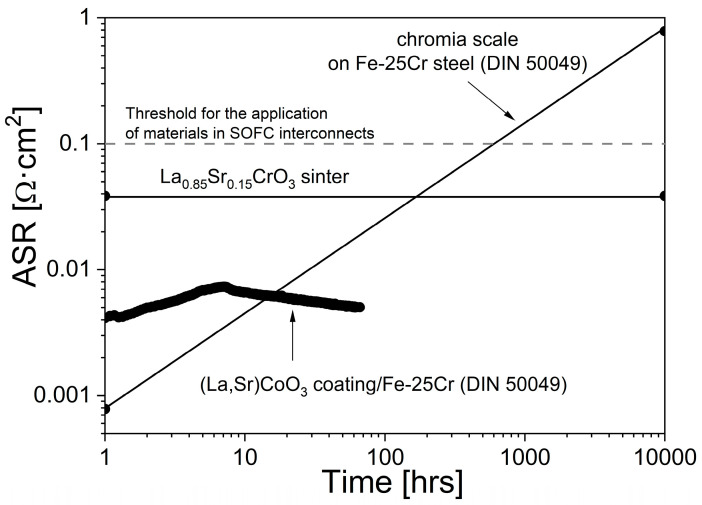
Area-specific resistance vs. time for (La,Sr)CoO_3_-coated and uncoated Fe-25Cr steels and for the ceramic (La,Sr)CrO_3_ at 1073 K.

Figure 13 shows a plot of the area-specific resistance vs. time for (La,Sr)CoO_3_-coated and uncoated Fe-Cr steels and for the ceramic (La,Sr)CrO_3_ at 1073 K.

The electrical resistance for the (La,Sr)CoO_3_ coating measured during oxidation in air for 70 h was about 5 mΩ·cm^2^. This figure also shows the calculated electrical resistance for the Cr_2_O_3_ scale on the uncoated Fe-25Cr steel based on the k_p_ value for the chromia scale growth [20,23] and the specific resistance of Cr_2_O_3_ [18], as well as for 0.5 cm-thick La_0.85_Sr_0.15_CrO_3_ [54]. This calculation indicates that the electrical resistance of the Cr_2_O_3_ scale on Fe-25Cr steel is higher than that of La_0.85_Sr_0.15_CrO_3_ after less than about 600 h in air at 1073 K, and thus, it should be used for an SOFC interconnector in composite form. The lower value of resistance for the (La,Sr)CoO_3_-coated Fe-25Cr steel, about 5 mΩ·cm^2^ in comparison with 35 mΩ·cm^2^—a characteristic value for commercial oxide ceramic interconnects—proved the possibility of applying it in fuel cell constructions [12].

Formation of the strontium–chromium oxide, SrCrO_4_, exhibiting better electrical conductivity than pure Cr_2_O_3_ [12] and being in contact with the chromia scale, results in an advantageous constant value of electrical conductivity of the whole Fe-25Cr/La_0.6_Sr_0.4_CoO_3_ layered system in SOFC cathode operating conditions.

Vaporisation of chromia, mainly constituting the oxide scale on ferritic steel at high temperatures in an operating SOFC, and the resulting formation of volatile chromium(VI) compounds have a harmful effect on the cells by tainting them, especially the cathode [14,15].

So it was essential to check the ability of the La_0.6_Sr_0.4_CoO_3_ coating to limit the emission of volatile chromium compounds from the Fe-25Cr steel substrate, which was determined by measuring the evaporation rate of Cr. The respective measurements were also conducted on the surface of the uncoated Fe-25Cr steel.

**Figure 14:**
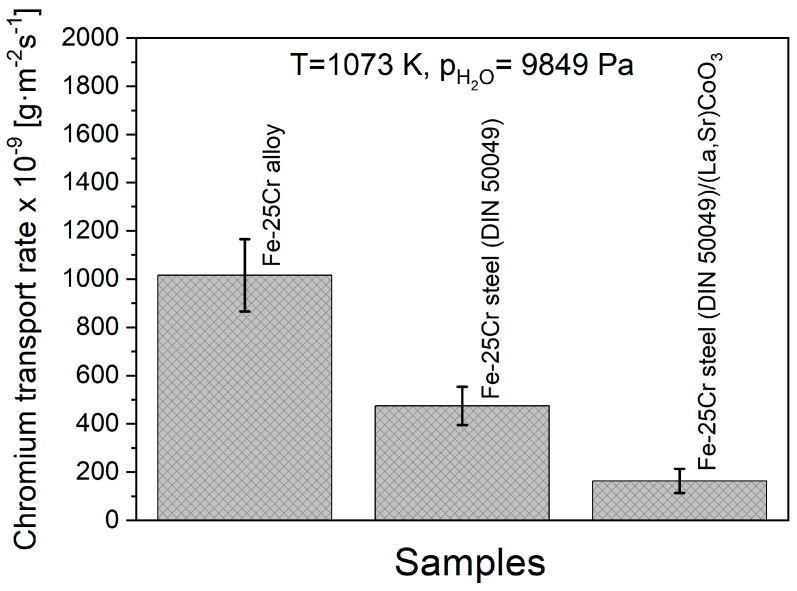
Chromium transport rate determined for Fe-25Cr alloy, Fe-25Cr steel, and Fe-25Cr steel coated with (La,Sr)CoO_3_. Test temperature: 1073 K. Test atmosphere: humidified air.

Figure 14 shows a comparison of the results of the rate of chromium vaporisation from the surface of the coated and non-coatedFe-25Cr steel oxidised in advance. This diagram also includes the results of the mentioned tests in relation to the Fe-25Cr non-commercial model alloy.

The highest value of chromium vaporisation rate (1.02 × 10^−6^ g·m^−2^·s^−1^) was noticed for the uncoated Fe-25Cr non-commercial model alloy. It formed pure chromia scales during high-temperature oxidation. The commercial Fe-25Cr steel (DIN 50049) was characterised by half the mass loss of the tested sample in comparison to the non-commercial alloy.

The explanation for such a difference in the measured rates of vaporisation is that the commercial one additionally formed a Mn_1.5_Cr_1.5_O_4_ spinel layer on the top of the chromia scale (Figure 6b).

According to Konycheva et al. [55], the chromium vaporisation rate from Crofer 22 APU during oxidation at 1073 K in air was 1/3 of the rate of vaporisation from the ODS alloy oxidised in the same oxidation conditions. Formation of a continuous (Mn,Cr)_3_O_4_ spinel layer at the gas/chromia scale interface on Crofer 22 APU accounted for the rate difference.

As demonstrated in Figure 14, as well as in the literature [56,57,58], depositing protective coatings on ferritic steels allowed for a more effective decrease (compared to steel without surface modification) in the chromium emission level. Application of the Sr-doped lanthanum cobalt coating in the layered system facilitates a decrease in the chromium vaporisation rate by around 65% compared to uncoated Fe-25Cr steel. As a result, it can be concluded that the suggested conductive La_0.6_Sr_0.4_CoO_3_ layer will play a protective role in the construction of metallic interconnectors for solid oxide fuel cells.

Since the target application of the investigated Fe-25Cr steel/(La,Sr)CoO_3_ coating layered system is the construction of SOFC stacks, the effect of the proposed modification on the electrochemical properties of the La_0.6_Sr_0.4_Co_0.2_Fe_0.8_O_3-δ_ (LSCF) or La_0.6_Sr_0.4_CoO_3-δ_ (LSC) cathode materials operating in the electrochemical cell mode also has to be taken into consideration and will be investigated in the future.

## 4. Conclusions

Fine La_0.6_Sr_0.4_CoO_3_ ceramic powder was produced via EDTA gel processes and applied screen printing technology to obtain thick films of a desired composition on Fe-25Cr steel plate substrates. Dense single-phase (La,Sr)CoO_3_ sintered compact was obtained from green bodies via 12 h of free sintering in air at 1473 K. This sinter consisted of rhombohedral-phase grains. Its electrical conductivity measured at 1073 K in air was ~144 S·cm^−1^. The cross-sectional SEM-EDS, TEM-SAD, and XRD investigations of the Fe-25Cr steel covered with (La,Sr)CoO_3_ after oxidation in air at 1073 K confirmed the formation of the compact, ceramic coating built of the LSC phase and separated from the steel substrate by the intermediate oxide layer at the coating/steel interface, formed during high-temperature oxidation reactions. This inner layer consists of several precipitations of La_2_O_3_, SrCrO_4_, and Co_3_O_4_, as well as thin and continuous layers of different compositions, including SrCrO_4_ and Cr_2_O_3_. As a result of the chemical interaction of the chromia scales with the (La,Sr)CoO_3_ coating, due to the diffusion of Sr from the coating to Cr_2_O_3_ grown on the steel surface at the Fe-25Cr/(La,Sr)CoO_3_ interface, a thin, continuous layer of SrCrO_4_ at the Cr_2_O_3_/(La,Sr)CoO_3_ border is formed. In situ measurements of the electrical resistance of the steel/La_0.6_Sr_0.4_CoO_3_ layered system during oxidation yielded an approximately constant value of about 5 mΩ·cm^2^ for 70 h, which is significantly smaller than the ASR value of 100 mΩ·cm^2^ allowable for SOFC interconnectors. Measurements of the formation rate of volatile Cr compounds for the Fe-25Cr steel (DIN 50049) without any modification and after the deposition of (La,Sr)CoO_3_ coating in a flowing air/H_2_O mixture at 1073 K demonstrated that this coating may serve as a barrier that effectively prevents the formation of volatile chromium compounds. The obtained results confirmed the usefulness of the Fe-25Cr ferritic steel (DIN 50049) coated with the La_0.6_Sr_0.4_CoO_3_ thick layer by screen printing as a potential construction material for metallic interconnectors for planar solid oxide fuel cells (SOFCs).

## Figures and Tables

**Table 1:** Chemical composition of the steel used in the present investigations.

Chemical Composition [mass%]								
Fe	Cr	Mn	Si	Ni	C	P	S	Ti
73.347	24.55	0.28	0.74	0.99	0.04	0.03	0.013	0.01

**Table 2:** Rheological parameters of (La,Sr)CoO_3_ powder after calcination at 1273 K for 25 h in air.

BET Surface Area [m^2^·g^−1^]	BET Mean Grain [µm]	Powder Characterising Parameters [µm]
14.68 ± 0.13	0.06	Surface weighted mean D[3.2] = 0.109 µm Volume weighted mean D[3.2] = 0.229 µm 1st mode: 9.73% for 0.12 µm 2nd mode: 3.97% for 0.72 µm Dv10 = 0.062 µm Dv50 = 0.120 µm (median) Dv90 = 0.678 µm

**Table 3:** Apparent density, relative density, and total porosity for the La_0.6_Sr_0.4_CoO_3_ compact sintered at 1473 K in air for 12 h.

Apparent Density [g·cm^−3^]	Relative Density [%]	Total Porosity [%]
6.36 ± 0.09	96.58	3.42

## Data Availability

The original contributions presented in the study are included in the article, further inquiries can be directed to the corresponding author.
